# Archaeology meets environmental genomics: implementing sedaDNA in the study of the human past

**DOI:** 10.1007/s12520-024-01999-2

**Published:** 2024-06-28

**Authors:** Kadir Toykan Özdoğan, Pere Gelabert, Neeke Hammers, N. Ezgi Altınışık, Arjen de Groot, Gertjan Plets

**Affiliations:** 1 Department of History and Art History, Utrecht University, Drift 6, Utrecht, 3512 BS Netherlands; 2 Animal Ecology, Wageningen Environmental Research, P.O box 47, Wageningen, Gelderland 6700 AA The Netherlands; 3 Evolutionary Anthropology, University of Vienna, Djerassiplatz 1, Vienna, 1030 Austria; 4 Human Evolution and Archaeological Sciences (HEAS), University of Vienna, Djerassiplatz 1, Vienna, 1030 Austria; 5 Environmental Archaeology, ADC ArcheoProjecten, Nijverheidsweg-Noord 114, Amersfoort, Utrecht, 3812 PN Netherlands; 6 Human-G Laboratory, Department of Anthropology, Hacettepe University, Ankara, 06800 Türkiye

**Keywords:** sedaDNA, Ancient DNA, Archaeogenomics, Ancient metagenomics, Bioarchaeology, Environmental archaeology

## Abstract

Sedimentary ancient DNA (sedaDNA) has become one of the standard applications in the field of paleogenomics in recent years. It has been used for paleoenvironmental reconstructions, detecting the presence of prehistoric species in the absence of macro remains and even investigating the evolutionary history of a few species. However, its application in archaeology has been limited and primarily focused on humans. This article argues that sedaDNA holds significant potential in addressing key archaeological questions concerning the origins, lifestyles, and environments of past human populations. Our aim is to facilitate the integration of sedaDNA into the standard workflows in archaeology as a transformative tool, thereby unleashing its full potential for studying the human past. Ultimately, we not only underscore the challenges inherent in the sedaDNA field but also provide a research agenda for essential enhancements needed for implementing sedaDNA into the archaeological workflow.

## Introduction

Ancient DNA or shortly *aDNA* refers to all types of DNA fragments that can still be retrieved from organisms that once lived. This opens a wide range of new possibilities to answer historical and archaeological questions. Indeed, the use of aDNA in archaeology has exploded in recent years. Yet, from the first ancient DNA study from human remains in the 1980s (Pääbo 1985) the interest of the archaeological community and most of the resulting studies has been focusing on the DNA of humans and other hominins, to investigate human evolution, ancestry and/or migration. The same methods can, however, also be applied to explore similar questions for other taxa.

Meanwhile, molecular ecologists discovered that DNA from e.g. flora, fauna, and microbes can not only be extracted from organic remains, but to some extent also from environmental contexts. While such *environmental DNA* (eDNA, see Table 1 for a glossary of terms) from contemporary organisms might be preserved in water (Thomsen et al. 2012), soil (Taberlet et al. 2012) and air (de Groot et al. 2021), eDNA in an archaeological context is typically recovered from sediments (either terrestrial or aquatic). In such cases, it is referred to as sedimentary ancient DNA or *sedaDNA*. SedaDNA samples hold genetic information of all living organisms that have interacted with a given layer or context. Therefore, sedaDNA has been embraced in paleoecology as one of the main methods for reconstructing past environments.

However, in archaeology, sedaDNA has mostly been used to detect extinct hominins and large prehistoric mammals (Gelabert et al. 2021; Parducci et al. 2017; Vernot et al. 2021; Wang et al. 2021). We thus argue that by leaving biodiversity assessments, especially from more recent periods, largely untouched, the archaeological community has yet to start making use of the full promise that sedaDNA has to offer. Compared to traditional archaeological methods, sedaDNA can classify a broader variety of taxa present in a sample, potentially at a drastically reduced cost.

Before sedaDNA can become a standard method in archaeology, concerted action is required. The sedaDNA field still is in continuous development, both with respect to wet lab protocols and the construction of tailor-made bioinformatic approaches to process the typically big amounts of genomic data produced per sample. Both developments require highly specialized skills and as a result, debates on advantages and disadvantages of sedaDNA applications are highly technical, which makes the field difficult to explore for archaeologists, perhaps even more so than any new method in archaeology. Moreover, it is highly tempting to become enchanted with the technique and overestimate its (current) potential. If we want to avoid the boom-and-bust cycle typical for many methods (Jones and Bösl 2021; Lebrasseur et al. 2018) we need to collectively develop a research agenda. To make sedaDNA a cornerstone in archaeology, we need to bridge the gap between on the one side archaeology and on the other side (ancient) environmental metagenomics.

Based on a structured literature review, the authors of this paper will outline the fundamental principles of sedaDNA and their successful applications. We then identify on which fronts it can contribute to fundamental archaeological research questions. We identify its greatest contribution to be to those discussions concerning livelihood practices, health, domestication, environmental reconstruction and (human) mobility. Subsequently, we will offer a research agenda highlighting the needed developments and approaches for efficiently implementing sedaDNA into archaeological research. We argue that sedaDNA can become a key instrument in the toolkit of any archaeologists if disciplinary-specific developments are made concerning sampling, data generation, reference data, ethics, open science, and comparison with established archaeobotanical and –zoological techniques.

**Table 1:** Common terms used in sedaDNA studies

Term	Meaning
**Ancient DNA (aDNA)**	DNA fragments obtained from fossils and ancient sediments
**Environmental DNA (eDNA)**	DNA fragments obtained from environmental sources
**Ancient environmental DNA (aeDNA)**	Ancient DNA obtained from ancient environmental sources
**Sedimentary ancient DNA (sedaDNA)**	Ancient DNA obtained from ancient sediment samples
**Paleogenomics**	The scientific field of reconstructing and analyzing ancient genomic material
**Metagenomics**	The study of genetic material, recovered from samples containing multiple organisms.
**Ancient metagenomics**	Metagenomics of ancient DNA samples
**Permafrost**	Surface materials (e.g. soil, subsoil) that remain consistently frozen regardless of seasonal variations.
**Pathogen**	A general term for microorganisms that cause diseases
**Mitochondrial DNA (mtDNA)**	Maternally inherited DNA that is present in a separate organelle, i.e. mitochondria, rather than nucleus of the cell
**Phylogenetic tree**	A tree-shaped graph that shows evolutionary relationship between organisms
**Polymerase chain reaction (PCR)**	A commonly method used for enzymatic amplification of DNA out of cell
**Shotgun sequencing**	A method for sequencing random fragments from a DNA library
**Target enrichment / target capture**	A laboratory method for selecting DNA fragments belonging chosen taxa in a DNA library
**Contamination**	Undesired DNA fragments from outside the sample, mostly modern DNA
**Authentication**	Determining whether the recovered DNA is ancient or not
**Damage pattern**	Specific chemical alterations occurred in DNA molecules in the post-mortem period that are used for authenticating ancient DNA
**Cross-contamination**	Contamination occurred between samples

**Fig. 1:**
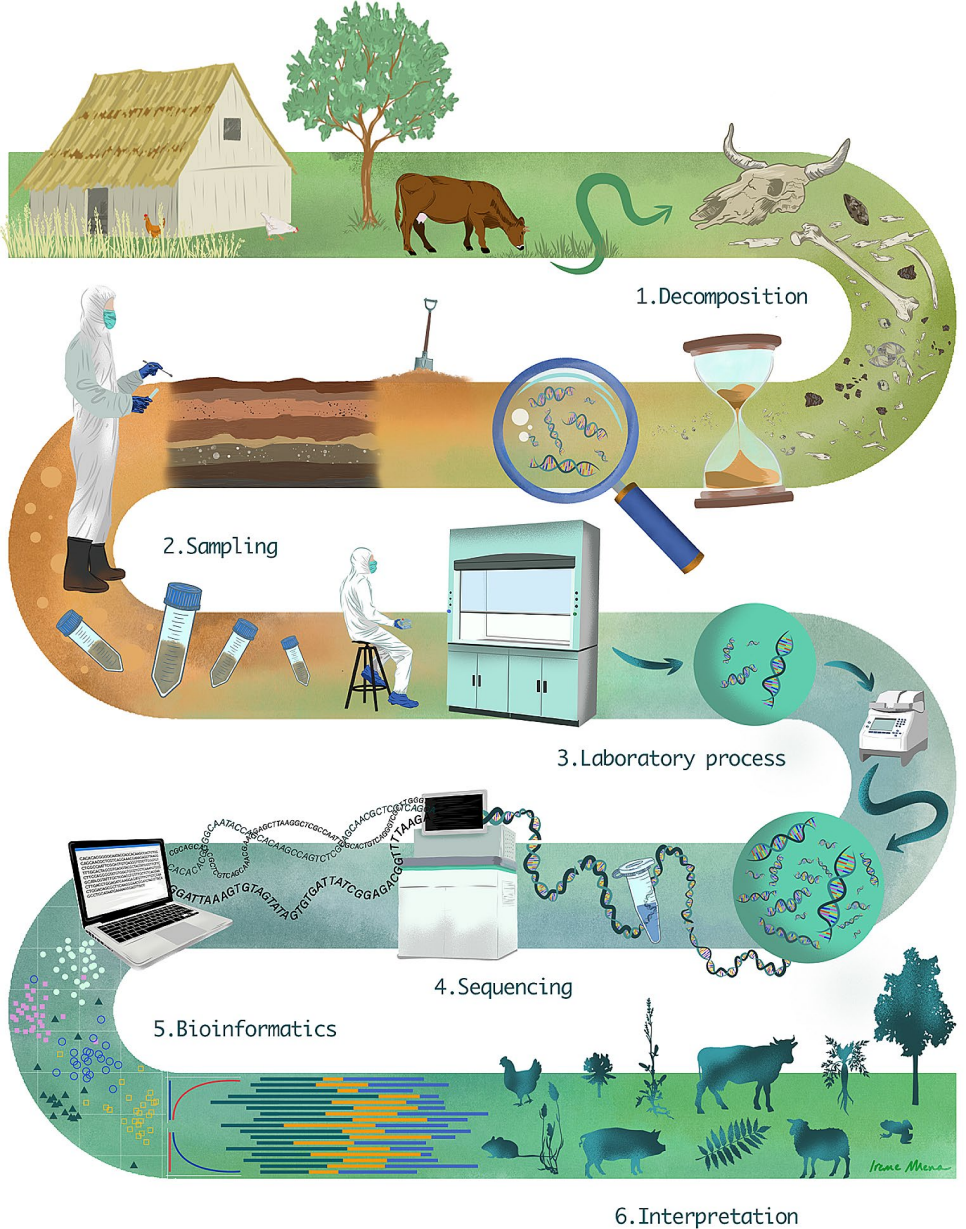
An illustration of basic sedaDNA workflow for archaeologists’ toolkit

## Current approaches and applications of sedaDNA

### Workflow

In most paleogenomics research so far, aDNA is extracted directly from larger preserved organic artifacts and ecofacts such as human or animal bones, and macro-botanical remnants or, in well-preserved contexts (e.g. permafrost), soft tissues of organisms (Maixner et al. 2016). In contrast, aDNA contained in a sediment sample consists either of free molecules bound to minerals in the soil (Kjær et al. 2022) or is contained in micro-remains or coprolites (Massilani et al. 2022). Due to the natural decomposition of organic materials, sedaDNA is damaged, degraded, fragmented, and intermixed (Orlando et al. 2021). Furthermore, a single ancient sediment sample most likely contains ancient DNA from a broad range of species, animal, vegetal, bacteria and fungi. These characteristics heavily determine and restrict the methodological workflow for sedaDNA analysis.

Nevertheless, the basic workflow for analysis of sedaDNA follows the same basic steps as for other types of aDNA. This workflow (Fig. 1) consists of strict protocols and workflows consisting of (1) sampling, (2) laboratory-based data generation, and (3) bioinformatic processing (see Fig. 1). Yet the exact tools used in every step overlap to some extent, but also differ in various aspects from a typical workflow for e.g. human aDNA. Moreover, multiple protocols are available for each step. As choices in this respect have direct implications for the types of questions that are tackled, below we first outline the most important features of a typical state of the art sedaDNA workflow.

#### Sampling

Like for any aDNA-based study, avoiding the influence of contemporary DNA as well as cross-contamination with actual ancient DNA from other contexts or adjacent soil layers is key whenever working with sedaDNA. While there are protocols throughout the whole workflow to prevent, detect and filter out contamination (e.g. (Dabney et al. 2013) such contamination should be avoided as much as possible already during sampling. SedaDNA samples are mostly taken either from the inside of soil sampling cores (Murchie et al. 2021) or directly from archaeological sections after removing the air-exposed top layers (Wang et al. 2021).

In both cases, sampling requires not only the use of sterile disposable materials and specialized protective clothing, but also adherence to strict rules with respect to careful handling of samples, and multiple rounds of cleaning tools and the workplace. To avoid irreversible contamination, this sampling process is normally carried out by trained DNA specialists. Yet, strict protocolization might yield opportunities for sampling by local personnel without a background in DNA analysis.

The selection of contexts is of key importance during sampling. Previous studies point to the potential impact of rooting on the contamination of archaeological deposits. This is especially relevant for more shallowly buried archaeological contexts (Ariza et al. 2023; Edwards et al. 2018). Also, studies on leaching in sedaDNA show that the degree of DNA mobility is influenced by a context’s sediment type and amount of organic matter (Andersen et al. 2012; Giguet-Covex et al. 2023). Finer clay soils leach less than e.g. coarser sand sediments. Organically rich multilayered contexts e.g. rich in feces can leach, while low-organic contexts such as e.g. buried walking surfaces influence lower layers less.

#### Data generation: laboratory process

DNA extraction from sedimentary samples happens in an ultra-clean (i.e., contamination-free) aDNA laboratory (Fulton and Shapiro 2019). Given the typically higher quantity and quality of DNA obtained from macrofossils, processing of such specimens along with environmental samples in the same clean lab can result in contamination risks, especially when sediment samples are screened for the presence of DNA of the same study organism. This results in a need for dedicated clean lab facilities for sedaDNA, which are currently very rare. Compared to macrofossils, sediments may contain relatively high amounts of substances that can disrupt later steps of the lab analysis. Such ‘inhibitors’ include humic acids, heavy metals, and complex proteins, naturally occurring especially in organically rich sediments. A series of additional protocols have been developed to remove inhibitors during or directly after DNA extraction (Armbrecht et al. 2020; Murchie et al. 2021; Rohland et al. 2018; Slon et al. 2017; Wang et al. 2021). Nevertheless, inhibition may remain a problem and the success of further lab analysis may depend on the type of soil and context the sample originated from.

As sedaDNA samples typically contain the DNA of a community of organisms, a workflow that is capable of parallel identification of multiple taxa is imperative. Over the last decade, so-called ‘DNA metabarcoding’ has been the standard method for this purpose (see e.g. Armbrecht et al. 2020). This method is based on the multiplication via PCR amplification of one or more fragments of the genome of which the code is known to vary among the taxa studied. For all resulting copies of those ‘barcode regions’ the DNA code is then translated to a letter code in a digital file (a ‘sequence read’). This translation is done in parallel for all copies in a process called ‘high-throughput sequencing’. DNA metabarcoding secures data for informative parts of the and downstream data analysis is relatively straightforward.

However, a significant downside of this method is that available tools to discriminate sequence reads from modern and ancient taxa (see below) cannot be applied on products of metabarcoding. As a result, datasets produced by DNA metabarcoding can be screened for modern contaminants. While tools are available to assess whether detected taxa are either modern or ancient (see below), such tools cannot be applied to the products of the metabarcoding approach (Armbrecht 2020). While authenticity checks may be less crucial for paleoenvironmental studies, it is essential when questions revolve around patterns in presence and absence of a certain taxon across samples from different locations and time periods. A different method, referred to as ‘metagenomics’ is therefore more suited for archaeological studies. Metagenomics is based on the sequencing of all DNA fragments in the original DNA extract regardless of their position in the genome. This approach results in a much more complex dataset yet does allow an authenticity check for each single taxon.

The untargeted data collection via metagenomics may require a deeper sequencing (i.e., collection of DNA codes for a larger subset of fragments from the DNA extract) to gather sufficient data for parts of the genome that are discriminatory among species or genetic variants within species. To reduce costs and/or enhance data volume especially for more detailed studies at the level of e.g., populations, strains or individuals, new approaches are developed for target enrichment, e.g. enrichment of the DNA pool for discriminatory fragments prior to sequencing (Furtwängler et al. 2020; Nichols et al. 2019).

#### Data processing: Bioinformatics

Bioinformatics, which represents the analytical step of sedaDNA studies, is a crucial step, especially now that advanced sequencing and extraction techniques are yielding vast amounts of data. Bioinformatic tools help researchers to (1) detect contamination, (2) perform the authentication, and (3) taxonomic classification. An aspect of bioinformatics, specific to metagenomic analysis as a tool for biodiversity screening, is the assignment of DNA sequences to specific taxa (species, or higher taxonomic levels). This process requires matching each individual sequence read against databases containing reference sequences of known taxa. Different bioinformatic tools have been published that automate this process (Hübler et al. 2019; Pochon et al. 2023; Yates et al. 2021). Further phylogenetic analysis using more focused reference datasets may be required to identify taxa with higher resolution (e.g. identification to species level after a preliminary match at family level).

Informatic techniques centered on the comparative analysis of multiple taxa, that integrate robust statistical discrimination methods, emerge as the most dependable and effective means to assess the presence of taxa (Vogel et al. 2023). After determining which organisms are present in a sample and authenticating them, it is possible to do further deeper analyses on the sequences of a selected taxon, depending on the quantity and quality of the recovered data. For instance, the co-occurrence of certain organisms can be used as an indicator of environmental dynamics (Wang et al. 2021), or the population history of a species from a specific site (Vernot et al. 2021) may be determined.

When high-quality genomes are available for particular species in a sedaDNA sample, combined with the tools offered by population genetics, it is possible to explore not only the presence/absence of species but also profound changes in genome structure and function which sheds light on the underlying evolutionary pressures.

### Research design and ethics

In recent years, ethical concerns within the field of aDNA research have prompted questions regarding privacy and data sovereignty, extending beyond human remains to include sedaDNA (Lewis et al. 2023). These concerns have significant implications, potentially impacting community assertions regarding treaties, repatriation, territorial disputes, and legal proceedings. Many indigenous communities maintain deep ontological connections to their ancestral lands, defined by an understanding of ecology that is not defined by the sharp distinctions between the realm of nature and humans (Bardill et al. 2018; Kowal et al. 2023). Traditional knowledge of plants, animals and environment can hold similar cultural value as human history and are often interwoven (Nabokov 2002; Swiderska et al. 2022).

Engagement with these communities not only mitigates complexities but also presents opportunities to integrate their oral and written histories, enriching research hypotheses (Lewis et al. 2023; Wagner et al. 2020). However, ethical considerations must extend beyond mere consultation with directly or indirectly affected individuals or research practices in colonial contexts. Disparities in funding and research infrastructure between the Global North and South often result in inequities, limiting the participation of researchers from the latter (Ávila-Arcos et al. 2022; Somel et al. 2021).

Collaborations with local researchers in sampled countries frequently employ superficial involvement strategies, hindering capacity building and technology transfer while perpetuating diversity deficits, a phenomenon often termed ‘helicopter science’ (Ávila-Arcos et al. 2022). Recent initiatives have yielded guidelines aimed at mitigating these challenges, emphasizing the importance of proactive engagement with local communities and researchers during the research design phase. Extensive discussions in literature have explored the challenges and potential solutions associated with such collaborations and research designs (e.g. Ávila-Arcos et al. 2022; Bardill et al. 2018; Kowal et al. 2023; Lewis et al. 2023).

### Current applications of sedaDNA: the influence of sediment type

Sediment is a very broad term, covering a wide range of source materials for isolation of sedaDNA found on the earth’s surface. For example, sediments can be terrestrial or aquatic (e.g. lake, river, or sea bottoms), can be permanently frozen (permafrost soils) or exposed to wide temperature ranges (e.g. dessert soils) and can be primarily mineral or exist mostly of organic materials (peat soil or some types of middens). SedaDNA research to date has focused mainly on particular sediment types (see Table 2 for a selection of recent publications per type). This focus was based on both specific research objectives, as well as practical consideration with respect to sample quality.

The relatively large body of studies that used sedaDNA for palaeoenvironmental research has mostly focused on aquatic sediments and permafrost sediments. And for good reasons: deep lake or marine sediments, as well as permafrost, are mostly relatively well structured and undisturbed, allowing a detailed analysis of the changes in composition of past communities over time (Parducci et al. 2019) (Capo et al. 2021).Several studies showed that it is possible to secure and reconstruct plastid genomes from DNA fragments isolated from aquatic sediments, efficiently enough to perform population genomic analyses (Lammers et al. 2021; Wang et al. 2021). Microbial genomes have been reconstructed based on sedaDNA from frozen marine sediment layers dated up to 120,000 years ago (Liang et al. 2021) and recent studies showed that DNA could be preserved at least 2 million years in permafrost, provides a unique opportunity to reconstruct ancient environments and uncloak alterations in floral and faunal communities starting from the Late Pliocene (Kjær et al. 2022).

Lake sediments may contain the DNA of animals and plants living in or above the water and organisms living in the surroundings, blown in by the wind or washed into the lake with river or rainwater. This yields information on the surrounding terrestrial ecosystems and potentially the human impact thereon (e.g. ter Schure et al. 2021), but also on human activity in for instance nearby lake settlements (e.g. Brown et al. 2022; Hudson et al. 2022) thus providing a valuable means to study archaeological questions. Meanwhile, sedaDNA has to a minor extent also been sampled directly from archaeological sites. Such studies mainly made use of relatively undisturbed sediments in caves and rock shelters to examine human presence and evolution (e.g. Slon et al. 2017; Vernot et al. 2021). While sedaDNA could be collected alongside any retrieved bone (thus limiting the need to damage the bone), human ancient DNA can in some cases also be retrieved from soil in the absence of bones, potentially tremendously enlarging sample sizes and spatiotemporal coverage of future population genetic studies. The same, in principle, applies to other faunal as well as floral taxa. For example, evidence of wolf and bison has been retrieved via sedaDNA from 25,000 years old cave sediments (Gelabert et al. 2021). Cave environments experience relatively stable climatic conditions, which may be advantageous for DNA preservation. A much smaller number of studies extracted sedaDNA from sediments collected at open-air archaeological sites and the potential of this sample type thus largely remains unknown. The few studies that tried mainly dealt with sediments containing organic-rich waste, such as latrines (e.g. Søe et al. 2018).

**Table 2:** Examples of articles that used various types of sediments as aDNA source

Sources of sedaDNA	Literature
**Lake sediment**	Ahmed et al. 2018; Crump et al. 2021; Graham et al. 2016; Hebda et al. 2022; Lammers et al. 2021; Liu et al. 2021; Moguel et al. 2021; Parducci et al. 2019; Pedersen et al. 2016; Rijal et al. 2021; Schulte et al. 2021; Wang et al. 2017, 2021
**Marine sediment**	Armbrecht et al. 2020, 2022; Gaffney et al. 2020
**Permafrost**	Kjær et al. 2022; Liang et al. 2021; Murchie et al. 2021, 2022; Wang et al. 2021
**Swamp forest sediment**	Dommain et al. 2020
**Rock shelter sediment**	Braadbaart et al. 2020
**Cave sediment**	Gelabert et al. 2021; Massilani et al. 2022; Pedersen et al. 2021; Slon et al. 2017; Vernot et al. 2021; Zavala et al. 2021; Zhang et al. 2020
**Archaeological sediment (incl. latrine, pond, well, midden)**	Boivin et al. 2024; Borry et al. 2020; Hudson et al. 2022; Moore et al. 2020; Sabin et al. 2020; Seersholm et al. 2016; Søe et al., 2018; Tams et al. 2018
**Aeolian sediment**	Azua-Bustos et al. 2019; Wygal et al. 2024; Yap et al. 2021

## Added value of sedaDNA in archaeology

Within the subfields of geoarchaeology, zooarchaeology, and archaeobotany, ecofacts are used to tackle fundamental archaeological research questions. Most studies to date use macro- or microscopic visual classification to identify past environments, species, and subsistence practices. Consequently, these methods are time-consuming and costly. Furthermore, morphological data is sometimes inadequate to distinguish closely related species. The principles of sedaDNA and the first trials of the method on archaeological contexts highlight metagenomic approaches that have the potential to transform those subfields. Yet, like any method, sedaDNA has limitations and visual-based inspection of ecofacts may yield more or better information in some cases. Therefore, the potential of sedaDNA in comparison to other ancient DNA sources (i.e., macro remains) or conventional archaeological methods depends on the type of question at hand and the type and resolution of data required to provide answers to that question. This potential is summarized in Table 3 and justified in more detail below.

**Table 3:** Potential of sedaDNA for answering different types of archaeologically relevant research questions (* ‘-’: limited, ‘+’: promising, ‘++’: significant)

Main questions	Specific topics	Conventional methods		Ancient DNA	
		Microscopic inspection (e.g. palynology)	Macroscopic inspection	Macro remains	Sediments
*What did they eat?*	Diet composition (plants and animals)	++	++	++	++
	Storage and transportation of food products	+	+	+	++
	Food preparation procedures	+	++	-	-
*How healthy were they?*	Detection of illness and disease	-	++	-	-
	Presence of pathogens and parasites (including zoonoses)	+	-	++	+
	Hygiene and healthcare indicators	++	++	++	++
*How did they domesticate their environment?*	Land management and spatial planning	++	+	++	++
	Agricultural practices	++	+	++	++
	Usage of wild animals (hunting and gathering)	-	++	++	++
	Domestication practices	-	++	++	+
	Effects on wild populations and natural ecosystems	+	+	+	++
	Movement of domestic livestock and plants during migrations	++	++	++	++
	Interbreeding of domestic varieties	-	-	++	+
*What did the environment look like?*	Ecosystem status and complexity	++	-	-	++
	Indicators of climatic fluctuations through biodiversity	++	-	-	++
	Indicators of past landforms	++	-	-	++
	Indicators of natural disasters (e.g. volcanic eruptions)	+	-	-	+
*Who were they and where did they come from?*	Mobility of people during their lifetimes	-	-	-	-
	Individual and population level migrations	-	-	++	+
	Diversity within the population	-	-	++	+
	Relatedness of given individuals	-	-	++	-

### What did they eat?

Organic remains are important indicators to reconstruct past subsistence, diets and cultural preferences. Micro- and macroscopic methods based on morphological characteristics currently define archaeozoological and archaeobotanical research. Increasingly, isotope ratios are also used for reconstructing diets (DeNiro 1987; Hedges and Reynard 2007; Katzenberg 2008; Larsen et al. 2019; Richards et al. 2001).

While off-site contexts such as ponds, and ditches can provide important insights into the fauna and flora consumed during a period (Reitz and Shackley 2012) especially latrines, occupation layers and trash contexts as are often rich in organic remains and provide evidence about the consumption of plants and animals. For the study of animal products, macrofaunal assemblages remain the main source of information. Microscopic analyses of e.g. pollen, and phytoliths provide the most important line of evidence for plants (Larsen et al. 2019; Santini et al. 2022; Warnock and Reinhard 1992).

Ancient DNA analysis of micro and macro remains can increase the taxonomic resolution to which they can be identified (Hagan et al. 2020; Kuch and Poinar 2012; Maixner et al. 2021; Nodari et al. 2021; Oskam et al. 2012). Also, ancient DNA analysis of human remains can provide evidence for adaptations to consumption changes. For example, the presence or diversity of genes coding for certain enzymes may signal certain diets (Fan et al. 2016; Rees et al. 2020).

As ancient DNA theoretically can be recovered from almost any archaeological context containing organic material, sedaDNA can be an efficient way in identifying key biomarkers. It may provide a faster way to scan pollen deposits without first extracting pollen. Furthermore, sedaDNA can provide a high level of taxonomic detail as DNA extracted from ecofacts. It may especially be valuable to allow the identification of species of which micro- or macro-remains are notoriously absent from the archaeological records due to preservation conditions. Likewise, sedaDNA may help to identify unrecognizable fragments and remains of food products in storage vessels (Drieu et al. 2020).

While sedaDNA holds potential for consumption practices, its potential to answer questions on food preparation procedures is more limited. Here, biochemical techniques can make a difference (Hendy 2021). Furthermore, DNA cannot differentiate between different parts of the same species. Likewise, it is always likely a consumption plant is present in a context because its pollen was blown in from the surroundings (de Groot et al. 2021).

Contexts such as latrines that are less influenced by external factors and abundantly rich in organic remains are especially suited for sedaDNA research into consumption practices. Clearly, sedaDNA and archaeozoological/botanical studies are not mutually exclusive, but complementary. Not only can such methods provide final proof for the presence of a taxon, visual inspection also provides more final proof of culinary processing (Martín et al. 2014; Medina et al. 2012; Yravedra et al. 2012).

### How was their health?

Osteological approaches are essential in studying individual life histories. Macroscopic inspection of human remains enables us to identify diseases (Ortner 2011). Traumas and healing patterns give insights into the daily life of individuals and the social and medical institutions around them (Altınışık et al. 2022; Spikins et al. 2018). Dental inspection (development, caries, wear etc.) enables the reconstruction of oral health and dietary habits (Larsen et al. 2019).

While human remains are an important source, they are not often available due to preservation conditions and burial practices, hampering systematic analysis. Findings of ancient pathogens can provide additional information and can be recovered from sediments rich in human feces. These contain microfossil remains associated with bacteria and viruses such as fungi and parasites (Sabin et al. 2020; Warnock and Reinhard 1992). Eggs of intestinal parasites (e.g. helminths) are generally well preserved in latrines and coprolites and have been the focus of paleoparasitological research for decades (Reinhard et al. 1986). While techniques for extraction and examination have been refined, overlap in morphological characters often limits identification beyond genus level (Søe et al. 2018). The integration of techniques from genetics into paleoparasitology has advanced abilities to identify eggs extracted from latrines or coprolites to the species level (Loreille et al. 2001, Søe et al., 2015).

Recent exploratory studies have shown that sedaDNA has the potential to be a versatile method for the recovery of ancient parasite DNA from soil (Søe et al. 2018). Depending on DNA preservation, direct extraction of sedaDNA could be a cost-effective alternative for sieving eggs from the sediment. Although larger numbers of eggs can be inspected by sieving them from the sediment, metagenomic analysis of a small sediment sample may already yield a large diversity of soil- and meat-borne parasites (Søe et al. 2018).

Over the past decade dental calculus, bone tissues, and dentine have been subject to aDNA analysis for identifying ancient protists, and bacterial and viral pathogens (de-Dios et al. 2023; Enard and Petrov 2018; Greenbaum et al. 2019; Guzmán-Solís et al. 2021; Margaryan et al. 2018). However, ancient pathogen DNA from environmental sources remains understudied but presents itself as an important domain for future innovation (Malyarchuk et al. 2022).

### How did they domesticate fauna and flora?

A central research question in archaeology remains the domestication of fauna and flora. Macrofaunal and archaeobotanical remains have been used to distinguish domestic species from their wild counterparts and identify markers of domestication. Visual comparison has been central in pinpointing the origin of domestication events of different species (Price and Hongo 2020). Unfortunately, for many species, it is not always possible to distinguish domestic and wild species since they are still physically close to each other. In such cases, tracing domestication events by using ancient DNA from organic remains becomes a more reliable alternative. There have been numerous genetic studies to detect evolutionary splits of wild and domestic species, as well as hybridization events afterward (Bergström et al. 2022). However, these ancient DNA studies still require well-preserved macroscopic organic remains.

Here, sedaDNA has the potential to fill in the gaps on the map between sites where such remains have been found, and thereby help researchers figure out the geographical distribution of domesticated species and, given sufficient quantity and quality of data, even the origins of those species. Likewise, sedaDNA may help to increase spatial coverage of historical distribution maps of wild species, as well as -on a more local scale- assessment of their presence in the surroundings of humans. A drawback of using sedaDNA for the detection of specific domesticated or wild variants of a taxon is that such variants may be hard to distinguish in case of low DNA yields from a certain taxon, quality or availability of references, postmortem damage (Atağ et al. 2022) and in some cases when samples have DNA from multiple closely related taxa.

### What was their environment?

Humans not only alter plants and animals, but human action is also prescribed by the environment. Biotic and abiotic factors define livelihood practices and are thus crucial for the interpretation of sites. A suite of faunal and botanic processes enables us to directly reconstruct both the biodiversity around a site and indirectly abiotic factors such as climate.

Certain archaeological contexts can also act as catchments where organic materials from the local or regional environment slowly accumulate (e.g. wells, deep canals, peat bogs or lakes). Microbotanical (pollen) and microfaunal (diatoms and insects) remain valuable sources for identifying key biomarkers that yield important insights into past landscapes and climate (Chevalier et al. 2020; Reitz and Shackley 2012).

Therefore, in many paleoenvironmental studies, sedaDNA has already been embraced as a fast, cost-effective and detailed alternative to palynology (Armbrecht et al. 2020; Gaffney et al. 2020; Murchie et al. 2021, 2022; Wang et al. 2021). These studies mostly use samples from permafrost or lake sediments. Archaeological contexts now used for environmental reconstruction through traditional methods can be used with the same methods.

However, cautionary selection and interpretation of contexts are needed as sedaDNA does not discriminate between material originating from wild populations and material originating from human practices, unless a clear signal of domestication is found. Furthermore, with traditional methods pollen can be quantified, providing insights into the dominance and divergence between species. Metagenomic analysis of pollen may provide trustworthy relative abundances among the observed taxa but fails to provide absolute densities, making it more difficult to make quantitative distribution assessments per species.

### Who were they and where did they come from?

Human mobility is proven to have been considerable throughout history (Lazaridis et al. 2014). While moving, people spread ideas, norms, and livelihood strategies. In the past, material culture was a central source for tracing human mobility. Important advances in the natural sciences have ensured that for the last two decades, DNA and isotopic ratios collected from human remains have been the main information sources for studying paleo-mobility (Bentley et al. 2003; Brettell et al. 2012; Duxfield et al. 2020; Pospieszny et al. 2023; Shaw et al. 2016).

As contemporary human DNA also enables us to map migrations routes and the genetic proximity of populations to each other (Gilbert et al. 2022), DNA from ancient individuals provides information about when and how population movements occurred. Therewith, answering important archaeological questions became possible, such as the distribution of certain languages or cultures (Fernandes et al. 2020; Wang et al. 2021).

Ancient human DNA from sediments might be advantageous when there are no human remains available because of several reasons, such as preventing destructive sampling, ethical concerns, the type of burial, or the absence of human remains in a site due to preservation levels. Despite the lower quality of data, studies have shown that it is possible to study the ancestry of individual genomes found from soil, enabling the performance of population genetics analyses with the absence of bone material (Gelabert et al. 2021; Pedersen et al. 2022). One of the limitations of this type of study, however, is that the recovered mtDNA or genomic data might originate from multiple individuals. This adds a layer of complexity to the population genetics analyses, which rely on the comparison of diverse genomic positions between individuals. A composite sequence that originates from multiple biological sources will present more variable positions than a real single genome, adding artificial divergence to the recovered consensus sequences (Gelabert et al. 2021).

### Summary: potential and position of sedaDNA in future archaeology

SedaDNA has the capacity to recover genetic material from unidentified taxa, or from taxa with scarce remains. Therefore, it presents itself as an essential method that can truly revolutionize our study of past consumption practices. Also, for the reconstruction of past landscapes and mapping of environmental change, it holds great potential. SedaDNA also has the potential to tackle other research questions, especially when certain organic remains are unavailable, have not been preserved, or are too costly for the excavators. However, sedaDNA is not the magic solution and single method that can solve all research questions.

Despite such limitations, we contend that sedaDNA has the potential to contribute to archaeology on three fronts:

1. Because of its versatility in cost-effectively identifying taxa from a given sample, it can add previously unseen details to archaeobotany, archaeozoology and geoarchaeology, and identify taxa which are impossible to classify due to taphonomy or lack of morphological distinctiveness.
2. Since it can map changes in consumption practices and the environment when zooming in on specific biomarkers, it can make existing models more fine-grained and provide us with detailed insights into changes over time.
3. It has the potential to straightforwardly map the absence or presence of key species, the technique presents itself as an ideal scanning or preliminary evaluation tool to assess the potential of a context or site for further analysis with traditional techniques.
4. Possibility to recover genomes of key species, including humans with absence of bones which provides a broader temporal and spatial distribution patterns of those taxa.

Clearly, sedaDNA is not to replace existing methods. Rather it should become part of the toolkit of those allied subfields studying organic remains. Perhaps its biggest potential lies in its role as a scanning method preceding further analysis.

## Towards a research agenda for sedaDNA in archaeology

With the decreasing sequencing costs, and improving laboratory and computational methods, sedaDNA is on track to become a standard method in archaeology. However, at this moment, sedaDNA remains an innovative application that has only been tested on a limited range of archaeological contexts and research questions. We need to ensure that we do not end up in a “hype cycle” where everybody is blindly enchanted by the potential of sedaDNA and the technique is widely applied without focus and criticism (Jones and Bösl 2021).

We argue that concerted collaborative research across international labs is needed to ensure sedaDNA becomes a trustworthy cornerstone in the study of the human past. Thus, a shared research agenda is imperative to focus methodological developments and thereby arrive at the right conditions so fundamental research questions in archaeology can be addressed with sedaDNA. We argue that for sedaDNA to become adopted as a standard method by archaeologists, five main topics require concerted attention.

### (1) Clean sampling strategies more easily integrated into the archaeological workflow need to be developed

 The current workflow requires trained DNA experts. However, archaeological (rescue) excavations often are ad hoc, making it difficult to plan and consistently sample those context rich in organics. Therefore, archaeologists would need to have the tools and knowledge at hand to sample those high potential contexts. This requires a standardized protocol executable for archaeologists that is both versatile and sterile enough for further extraction in the lab.

### (2) Increasing the data resolution for specific archaeological research questions

The level of detail in the data collected for archaeological purposes depends on the specific research questions. Before gathering data, it’s important to plan thoroughly based on the questions and available resources to make the process efficient. Different methods are used to collect data at various levels of detail (see 2.2.). While studying patterns in presence/absence of species or groups of species and the overall biodiversity of an environment, shotgun metagenomics is effective. On the other hand, the studies on broad patterns of change in ecosystem composition, combined with contexts with very low chance of leeching and disturbance (e.g. lake sediments) might be approached with metabarcoding. However, for a detailed study of a particular organism, targeted enrichment is preferred. Targeted enrichment, while detailed, requires prior knowledge of the genetic diversity of the organism being studied. Some well-studied species have ample genetic data available, making this process easier. However, for less studied organisms, designing capture probes for targeted enrichment can be difficult and may reduce the quality of the data. One long-term solution is to collect more genetic data over time from a wide range of organisms. In the short term, openly sharing designed capture probes along with research publications can help improve the quality of data for less studied organisms.

### (3) More comprehensive reference databases to increase the quality of taxonomic and evolutionary estimations

 Since the taxonomic classifications heavily depend on the reference genomes available, the lack thereof creates false positives and negatives during the analysis. To tackle it, more reference genomes from different taxa are needed. Existing initiatives beyond archaeology show that sequencing genomes of relevant species can increase the quality of bioinformatic species determination.

### (4) Fostering an open science perspective where raw data and methodological protocols are shared

Such an approach helps other scientists use similar methods for their own studies or helps them avoid double work for certain aspects of their studies. Next to those, when the shared data is raw and unprocessed sequencing data, it allows other scientists to re-use it for comparison or certain types of analyses that were not done during the actual study, due to different focus or a limited time. Once the raw data is shared it becomes a good resource for other scientists and contributes to the growth of the scientific field.

### (5) Multi-proxy comparisons between sedaDNA and other archaeological methods

 The comparison of results of sedaDNA with e.g., pollen analysis enables us to explore the limits and blind spots of sedaDNA. This information will help us in positioning sedaDNA in the archaeological workflow and thus solve the important question: at which stage can sedaDNA bring added value and be efficiently integrated into the current workflow.

### (6) Integrating ethical concerns in the research

 The research plan should be developed from an ethical perspective in collaboration with local stakeholders. International researchers must prioritize the needs of local collaborators by engaging them as equal partners in the research process. This necessitates including provisions for local capacity building, human resource management, and technology transfer within the research plan, particularly in collaborations with researchers from the Global South and underrepresented people in the field (see Sect. 2.2).

## Conclusion

SedaDNA has become an essential tool in the field of paleogenomics while its applications for archaeology remain limited. This article highlighted the vast potential of sedaDNA to address pressing archaeological questions. Especially, the research questions related to diets, origins, and environments of ancient human populations are yet to unleash this potential. However, it is evident that future studies need to overcome several key challenges. These are mainly about its applicability in archaeological workflows, data resolution and quality and comprehensive reference databases. Our primary goal is to facilitate a broader use and to unlock the full potential of sedaDNA in archaeological research. The optimization of sedaDNA’s use in archaeological research will provide newly found perspectives in the pursuit of a deeper understanding of the human past. Therefore, a concerted collaboration between sedaDNA labs is needed if it would become a cornerstone of the archaeological practice.

## Data Availability

No datasets were generated or analysed during the current study.
